# Effects of H3.3G34V mutation on genomic H3K36 and H3K27 methylation patterns in isogenic pediatric glioma cells

**DOI:** 10.1186/s40478-020-01092-4

**Published:** 2020-12-07

**Authors:** Tina Yi-Ting Huang, Andrea Piunti, Jin Qi, Marc Morgan, Elizabeth Bartom, Ali Shilatifard, Amanda M. Saratsis

**Affiliations:** 1 Department of Neurological Surgery, Northwestern University Feinberg School of Medicine, Chicago, IL 60611 USA; 2 Department of Biochemistry and Molecular Genetics, Northwestern University Feinberg School of Medicine, Chicago, IL 60611 USA; 3 Division of Pediatric Neurosurgery, Department of Surgery, Ann & Robert H. Lurie Children’s Hospital of Chicago, 225 E Chicago Avenue, Box 28, Chicago, IL 60611-2991 USA

**Keywords:** Pediatric high-grade glioma, Post-translational modifications, H3K36me3, Histone H3 mutations

## Abstract

Histone H3.3 mutation (*H3F3A*) occurs in 50% of cortical pediatric high-grade gliomas. This mutation replaces glycine 34 with arginine or valine (G34R/V), impairing SETD2 activity (H3K36-specific trimethyltransferase). Consequently, reduced H3K36me3 is observed on H3.3G34V nucleosomes relative to wild-type, contributing to genomic instability and driving a distinct gene expression signature associated with tumorigenesis. However, it is not known if this differential H3K36me3 enrichment is due to H3.3G34V mutant protein alone. Therefore, we set to elucidate the effect of H3.3G34V mutant protein in pediatric glioma on H3K36me3, H3K27me3 and H3.3 enrichment in vitro. We found that the doxycycline-inducible shRNA knockdown of mutant *H3F3A* encoding the H3.3G34V protein resulted in loss of H3.3G34V enrichment and increased H3K36me3 enrichment throughout the genome. After knockdown, H3.3G34V enrichment was preserved at loci observed to have the greatest H3.3G34V and H3K36me3 enrichment prior to knockdown. Induced expression of mutant H3.3G34V protein in vitro was insufficient to induce genomic H3K36me3 enrichment patterns observed in H3.3G34V mutant glioma cells. We also observed strong co-enrichment of H3.3G34V and wild-type H3.3 protein, as well as greater H3K27me3 enrichment, in cells expressing H3.3G34V. Taken together, our study demonstrates the effects of H3.3G34V mutant protein on genomic H3K36me3, H3K27me3 and H3.3 enrichment patterns in isogenic cell lines.

## Introduction

Pediatric high-grade glioma (pHGG) is the number one cause of cancer death in children, with a 5-year survival of less than 20%. This dismal prognosis is in large part due to an historical lack of understanding of its distinct biology and the presumption that pHGG is biologically identical to its adult counterpart, resulting in ineffective treatment. However, with the development of next-generation sequencing technologies to analyze rare tumor specimens, knowledge of pHGG biology significantly increased over the past decade. Somatic missense mutations in genes encoding Histone H3 isoforms, including *H3F3A, HIST1H3B* and *HIST1H3C,* were subsequently identified in up to 50% of supratentorial hemispheric pHGG, and 80% of pediatric diffuse midline gliomas (DMG), a form of pHGG in the thalamus or brainstem [11, 15, 20]. These mutations are associated with distinct tumor biology and poorer clinical outcome, and are now understood to play a role in pediatric gliomagenesis. As a result, determining the effects of these mutations on histone H3 function and regulation of gene transcription, in order to identify more effective therapeutic targets, is of great interest.

In hemispheric pHGG, somatic mutations of *H3F3A* (GGG to GTG) result in a glycine 34 to arginine (G34R) or valine (G34V) substitution on the histone H3.3 N-terminal tail, while *H3F3A* mutations in DMG result in lysine to methionine alterations (K27M or K36M). Several studies have focused on the effects of these mutations on global methylation, chromatin structure, and transcription regulation to promote tumorigenesis. For example, mutant H3.3K27M protein exhibits higher affinity for EZH2, a H3K27-specific lysine methyltransferase, compared to wildtype H3.3, resulting in EZH2 sequestration and preventing PRC2 from propagating transcriptionally repressive H3K27 methylation [14]. Mutant H3.3K36M protein inhibits H3K36-specific lysine methyltransferases, including NSD1, NSD2, and SETD2, reducing global H3K36 methylation [3, 12, 21]. In contrast, less is known about the epigenetic and tumorigenic effects of H3.3G34V/R mutations in pHGG. Several studies comparing H3.3G34V mutant and wild type cell lines suggest distinct epigenetic changes at H3K27 and H3K36 in association with H3.3G34V/R mutation, as well as alteration to DNA repair pathways leading to transcriptional upregulation, increased mutational burden and genomic instability [2, 10, 16]. However, as these studies did not use isogenic cell lines, the distinct mechanism by which H3.3G34V/R mutation exerts the observed changes is not clear. Therefore, we set to better elucidate the direct epigenetic effects of H3.3G34V mutation in pediatric glioma in vitro using isogenic cell lines. Here, we demonstrate changes in genomic enrichment of multiple chromatin marks after DOX-inducible knockdown of *H3F3A* in an H3.3G34V mutant pediatric glioma cell line, and H3.3G34V mutation transduction in wild-type astrocytes, providing insight on epigenetic effects of this mutation that promote tumorigenesis.

## Materials and methods

### Cell lines and culture conditions

Experiments were conducted using an established H3.3G34V mutant patient-derived pediatric high-grade glioma cell line (KNS42), and Histone H3.3 wild-type human astrocytes (NHAs, ScienCell #1800). KNS42 was obtained from Rintaro Hashizume (Northwestern University), it is well characterized as previously described [6]. KNS42 cells were maintained in EMEM (10009CV, Corning) with 5% FBS (16000-044, Gibco). NHA cells were maintained in high glucose DMEM (11995-065, Gibco) and 10% FBS, according to the cell line provider’s recommendation. Cells were grown in an incubator with 5% CO_2_ at 37 °C. All experiments were conducted in accordance to institutional protocols and approvals (NU IRB# STU00202063).

### H3.3G34V mutation induction and knock-down

Lentiviral delivery of a doxycycline-inducible RNAi vector targeted against *H3F3A* was transduced to KNS42 cells to knock down H3.3G34V protein expression. The vector contains the selectable markers of puromycin as well as a red fluorescent protein (RFP) reporter. Lentiviral vector-mediated doxycycline-inducible cDNA encoding a c.104G > T p.(Gly34Val) *H3F3A* mutation was transduced to NHAs in order to express H3.3G34V mutant protein. This vector, pUC57-Kan, contained a kanamycin resistant gene. A vector containing doxycycline-inducible *H3F3A* for wild-type H3.3 protein expression was used as negative control. All vectors were purchased from Genscript. A total of 250,000 cells from each cell line were transduced with lentivirus for 6 h, rinsed in PBS, then cultured in their respective media as described above. A second round of lentiviral transduction was performed 24 h later. After an additional 24 h, antibiotics were added at 2 µg/mL. Cells were then cultured in their respective media for three to 5 days to achieve desired confluency. For doxycycline-induced transduction conditions, doxycycline was added at 1:5000 every other day for 1 week (days one, three, five, seven), and on day eight cells were collected as a pellet for Western Blotting to confirm protein expressions, or crosslinked with 1% formaldehyde for ChIP-Seq (see below). Cells without doxycycline treatments were cultured and collected in parallel. Fluorescent imaging and flow cytometry were performed to select for cells with successful protein knockdown or transduced expression.

### Western blotting

Protein was extracted from cells using RIPA buffer (89900, Thermo Fisher Scientific). A total of 60 µg of protein (from whole cell extract) was separated by electrophoresis in a 4–15% precast protein gel (4561086, BioRad) and transferred to PVDF membranes. Blocking was subsequently performed with 5% non-fat milk in TBST, followed by incubation with anti-H3K27Ac antibody at 1:500 dilution (8173S, Cell Signaling Technology) overnight. After 5 washes with TBST, membranes were incubated with HRP-conjugated anti-Rabbit IgG antibody at 1:1000 (7074 Cell Signaling Technology) for 1 h. Pierce ECL Plus (32132, Thermo Fisher Scientific) was used to detect protein bands. Blots were then stripped (46430, Thermo Fisher Scientific) and re-probed with anti-total H3 primary antibody at 1:1000 dilution (14269S, Cell Signaling Technology) as a loading control. HRP-conjugated anti-Mouse IgG antibody (7076, Cell Signaling Technology) was used to detect total H3 signal. Densitometry analysis was performed with image J.

### Cell proliferation assay

Cell proliferation was measured by counting viable cells using the TC20 Automated Cell Counter (Bio-Rad). 3 × 10^5^ cells were seeded in cell culture dishes. At 3 and 7 days after seeding, cells were harvested, dissociated into single cell suspension, and stained with 0.4% Trypan Blue Solution (15250061, ThermoFisher) for 5 min before counting.

### Cell viability assay

Cell viability was assessed using the CellTiter-Glo Luminescent Assay (G7570, Promega). 3000 cells were seeded in 96-well plate. Measurements were taken 1, 3, 5, and 7 days after seeding. Reagent was diluted at 1:1 ratio with PBS to achieve optimal luminescent range. 100 µL reagent was added to cells in 100 µL media. The mix was incubated for 10 min at room temperature with gentle shaking, followed by luminescent measurement.

### Cell crosslinking and chromatin immunoprecipitation

For each immunoprecipitation, 30 million cells were crosslinked using freshly prepared 1% formaldehyde in complete cell medium for 10 min at RT, and subsequently quenched with 0.125 M glycine for 5 min. The cells were then rinsed twice in cold PBS, gently scraped from the plates and centrifuged in a 15 mL tube (Falcon) at 1350×*g* for 8 min at 4 °C. Crosslinked cells were either stored in − 80 °C or used immediately for chromatin immunoprecipitation, as previously described [14]. Briefly, crosslinked cells were resuspended in 10 mL buffer 1 for 10 min at 4 °C then centrifuged at 1350×*g* for 5 min at 4 °C. Pellet was resuspended in 10 mL buffer 2 for 10 min at RT then centrifuged at 1350×*g* for 5 min at 4 °C. Pellet was resuspended 1 mL buffer 3 and transferred to a 1 mL milliTUBE (520135, Covaris). Sonication was performed using the Covaris E220 ultrasonicator with the following parameters: 20% duty cycle, 175 PIP, 200 cycles/burst, for 8 min. After sonication, sample was centrifuged at 20,000×*g* for 15 min at 4 °C and supernatant containing chromatin was collected. 50 µL of chromain were de-crosslinked with elution buffer for 3 h at 65 °C and DNA was extracted using PCR purification kit (28104, Qiagen). Purified DNA were loaded in a 1.5% agarose gel to check for the fragment size (average range 200–500 bp). A total of 100 µL ChIP dilution buffer was added to the remaining sheared chromatin. A total of 10 µL of each sample was saved at 4 °C to serve as input. The remaining chromatin was incubated with primary antibody on a nutator overnight at 4 °C (refer to Table 1 for a list and dilution of antibodies used). The following day, 60 µL of 50% protein A/G agarose beads (sc-2003, Santa Cruz Biotech) were added to the samples and incubated for 3 h on a nutator at 4 °C. Agarose beads were pelleted by centrifugation at 2500×*g* for 1 min and washed with 1 mL of RIPA buffer four times followed by 1 mL of 50 mM NaCl in TE. The beads and the 10 µL input sample were resuspended in 200 µL elution buffer for 30 min at 65 °C then centrifuged for 2 min at 15,000×*g*. Supernatant was transferred into a new 1.6 mL tube and de-crosslinked overnight 65 °C. The following day, DNA was extracted with PCR purification kit (28104, Qiagen), and eluted to a final volume of 60 µL. A total of 45 µL of each sample and input were used for library preparation and subsequent sequencing steps. All additional reagents (Elution buffers 1, 2, 3, Elution buffer, 10× ChIP dilution buffer, RIPA buffer, and elution buffer) were prepared as previously described [14].

**Table 1:** List of antibodies used

Antibody	Company	Catalog #	ChIP	WB
H3.3WT	Millipore	09-838	10 µg	1:1000
K36me3	Homemade	na	30 µL	1:1000
G34V	RevMAb	31-1193-00	10 µg	1:1000
K27me3	Homemade	na	30 µL	na
Total H3	CST	14,269	na	1:1000
Anti-Rabbit IgG, HRP	CST	7074	na	1:5000
Anti-Mouse IgG, HRP	CST	7076	na	1:5000

*We would like the table to be inserted following the section “Cell Crosslinking and Chromatin Immunoprecipitation” of Materials and Methods

### Library Preparation and Next-Generation Sequencing

ChIP-Seq libraries were prepared with the KAPA Library preparation kit (KK8234, Kapa Biosystems) and NETflex DNA barcodes (514104, Bioo Scientific). A total of 10 µg DNA was used as starting material for input and immunoprecipitation samples. Libraries were amplified with a thermocycler for 13 cycles. Post-amplification libraries were size-selected at 250-45 bp in length using the Agencourt AMPure XP beads (A63881, Beckman Coulter). Libraries were validated using the Agilent High Sensitivity DNA Analysis Kit (5067-4626). ChIP-Seq libraries were single-read sequenced on the Illumina NextSeq 500 Sequencing System.

### ChIP-Seq data analysis

Reads were filtered using the FASTX-Toolkit suite and read quality was assessed with FastQC v0.11.5. After removal of duplicated reads, unique reads were mapped to human reference genome Hg38. ChIP-Seq reads were aligned with the ENCODE pipeline. Peaks were called with MACS2 v2.1.0 software with cutoff of *p *< *0.01*. Enrichment values were determined as log2(Normalized experimental read count—normalized input read count) in regions of interest in each ChIP specimen and corresponding input sample. Called peaks were annotated with HOMER v4.10 to the nearest gene. Active enhancers were defined as H3K27ac peaks excluded from the transcription start site (TSS) (2.5 kb upstream and downstream). ChIP-Seq reads density and data visualizations were generated using Deeptools v3.1.1. Additional functional pathways and upstream regulator analysis was performed on differentially enriched loci using Ingenuity Pathways Analysis software (Qiagen, Germantown MD).

## Results

### Lentiviral induced knockdown and expression of H3.3G34V

**Fig. 1:**
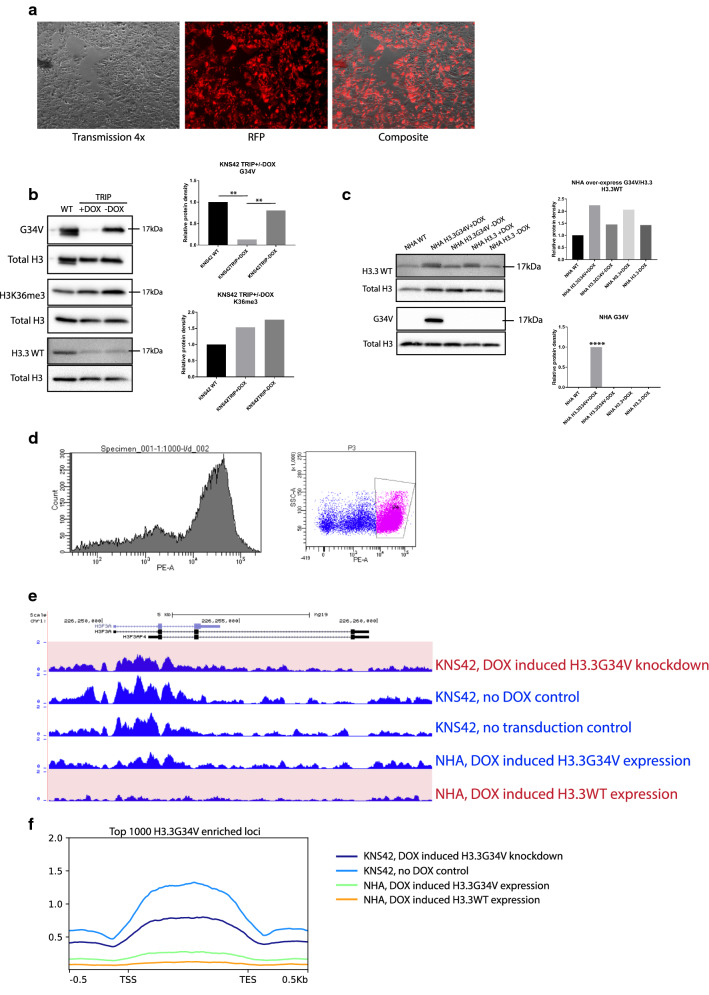
Genetic Modification of Histone H3.3 expression in Pediatric Glioma Cells and Astrocytes. **a** Expression of RFP reporter was confirmed in > 95% of Doxycycline-induced cells. **b** H3.3G34V expression was significantly reduced in lentiviral-transduced KNS42 cells treated with Doxycycline, compared to no Doxycycline and control conditions. **p < 0.01, ****p < 0.0001. **c** H3.3G34V protein was expressed in lentiviral-transduced NHAs treated with doxycycline, with no H3.3G34V expression observed in controls. **d** Flow cytometry was used to select the top 30% cells with H3.3G34V knockdown and knock-in for subsequent ChIP-Seq analysis. **e** Genome browser view of H3.3G34V enrichment at the *H3F3A* locus across cell lines studied. Tracks highlighted in red are those conditions without H3.3G34V expression. **f** Metagene profile of H3.3G34V enrichment in the gene body across the top 1000 most H3.3G34V-enriched genes in KNS42. Reduction of H3.3G34V enrichment is observed following doxycycline-induced knockdown (light blue versus navy blue lines). Greater H3.3G34V enrichment is also observed in NHA transduced for H3.3G34V expression (green line) compared to control transduced for H3.3WT expression (orange line)

To characterize the epigenetic effects of H3.3G34V mutant protein in pediatric glioma, we compared global methylation and Histone H3 enrichment patterns in isogenic cell lines. We used doxycycline-induced lentiviral delivery of genetic constructs designed to silence (shRNA) or express (cDNA) mutant and wild-type *H3F3A* in H3.3G34V mutant glioma cells (KNS42), and H3 wild type astrocytes (NHA). Using fluorescent imaging, we measured lentiviral RFP expression after DOX-induction to confirm vector expression (Fig. 1a). Western blot analysis of whole-cell extracts confirmed successful knockdown of H3.3G34V mutant protein in glioma cells treated with doxycycline, compared to those not treated with doxycycline and those transduced with *H3F3A* for wild-type H3.3 expression, with no significant reduction in H3K36 trimethylation across conditions (Fig. 1b). In turn, we observed DOX-induced expression of H3.3G34V mutant protein in cDNA transduced astrocytes, with no mutant protein detected in untreated cells, or in cells transduced with *H3F3A* (Fig. 1c). While we achieved significant overexpression of H3.3G34V mutant protein in astrocytes compared to control, the maximum level of mutant protein we could express in astrocytes is only 28.9% of the level observed in H3.3G34V mutant glioma KNS42 (Additional file 1: Figure S1A, B). Flow cytometry was used to select the top 30% cells with successful H3.3G34V knockdown and knock-in, for subsequent ChIP-Seq (Fig. 1d). We did not observe significant difference in cell viability (Additional file 1: Figure S1C) or proliferation (Additional file 1: Figure S1D, E) between KNS42 with and without doxycycline-induced H3.3G34 knockdown, nor between NHA overexpressed with H3.3G34V and H3.3 control. On ChIP-Seq analysis, we observed decreased H3.3G34V enrichment at *H3F3A* in KNS42 glioma cells after DOX-induced transduction of *H3F3A* shRNA, relative to KNS42 cells that were transduced without DOX induction, or not transduced (Fig. 1e). We also confirmed enrichment of H3.3G34V mutant protein at *H3F3A* in DOX-induced astrocytes, with no mutant protein in untreated NHAs or those transduced with wild-type *H3F3A* cDNA (Fig. 1e). Outside of *H3F3A,* metagene plots of H3.3G34V enrichment within the gene body for the top 1000 most H3.3G34V-enriched genes demonstrated reduction, but not complete elimination, of H3.3G34V deposition in KNS42 with DOX-induced knockdown compared to KNS42 cells that were transduced but without DOX induction. Higher H3.3G34V enrichment is also observed in NHAs transduced for H3.3G34V expression, compared to NHAs with only wild-type H3.3 expression (Fig. 1f).

### H3.3G34V Co-enriches with Wild-type H3.3

**Fig. 2:**
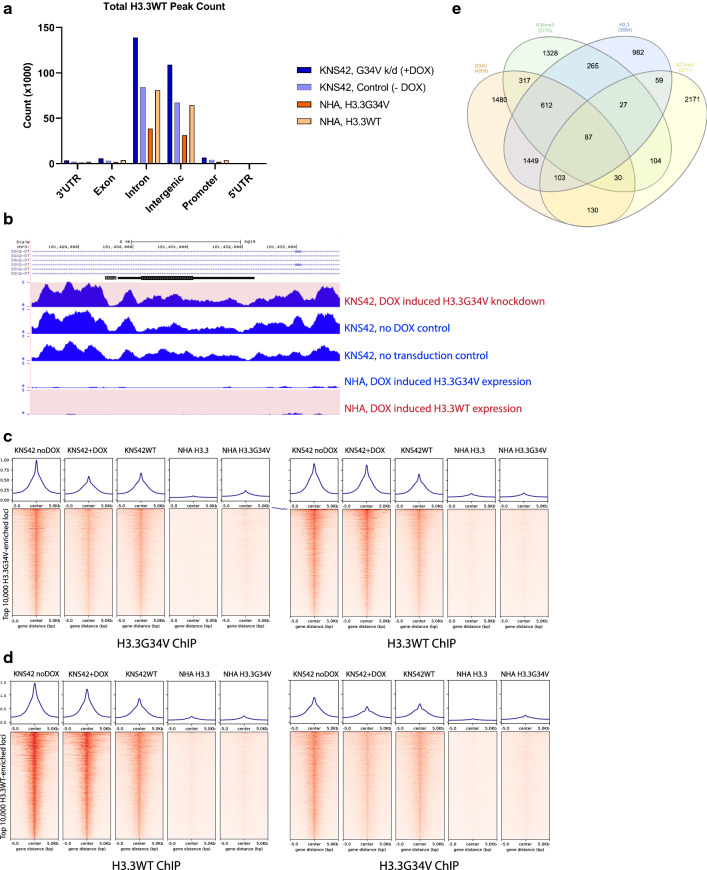
H3.3G34V Co-enriches With Wild-type H3.3. **a** Relative proportion of H3.3WT enrichment across gene elements with H3.3G34V expression or knock-down. **b** H3.3 enrichment at *SOX2* was greatest KNS42 cell s expressing H3.3G34V, compared to NHAs lacking H3.3G34V expression (red highlight) or after H3.3G34V transduction. **c**, **d** Co-enrichment of H3.3G34V and H3.3WT was observed across experimental conditions at the top 10,000 loci most-enriched for C) H3.3G34V, and D) H3.3WT in transduced KNS42 cells without DOX induction. **e** When the top 10,000 loci most enriched for H3.3G34V, H3.3 wild-type, H3K36me3 and H3K27me3 in transduced KNS42 cells without DOX induction are compared, greatest overlap is observed between H3.3G34V and wild-type H3.3 enrichments patterns, with 3041 common loci between these two groups

Overall, we observed greater H3.3 enrichment in cells expressing H3.3G34V mutant protein. Specifically, we identified 270,656 wild-type H3.3 peaks in glioma cells after H3.3G34V knockdown (6628 promoter, 144,735 gene body), and significantly fewer H3.3 peaks in KNS42 glioma cells with intact H3.3G34V expression (164,785 total, 4071 promoter, 87,770 gene body, Fig. 2a). The same trend was observed in astrocytes induced to express H3.3G34V (77,018 total peaks, 2019 promoter, 40,530 gene body) compared to controls (15,9235 total peaks, 3952 promoter, 84,746 in gene body, Fig. 2a). At genes previously reported to have greater WT H3.3 enrichment in H3,3G34V mutant tumors compared to WT, manipulation of H3.3G34V mutant protein expression had no effect on WT H3.3 enrichment (Fig. 2b). To further determine the effect of H3.3G34V expression on genomic enrichment of WT and mutant H3.3, we identified the top 10,000 loci most-enriched for H3.3G34V in transduced KNS42 cells without DOX induction (negative control), and compared H3.3G34V and WT H3.3 enrichment at these loci across experimental conditions. We detected co-enrichment of H3.3G34V and WT H3.3 in all glioma cell treatment conditions at these 10,000 loci most enriched for H3.3G34V (Fig. 2c), as well as at the 10,000 loci most enriched for WT H3.3 (Fig. 2d). When these loci are mapped to the nearest gene, H3.3G34V and WT H3.3 enriched loci have the most overlap, with 3041 shared loci between groups (Fig. 2e). Taken together, these data suggest that glioma H3.3G34V expression is associated with increased WT H3.3 enrichment throughout the genome, that WT H3.3 and H3.3G34V strongly co-enrich, and that loci most enriched by these proteins do not significantly change with H3.3G34V knockdown.

### H3.3G34V does not affect global H3K36me3 enrichment levels

**Fig. 3:**
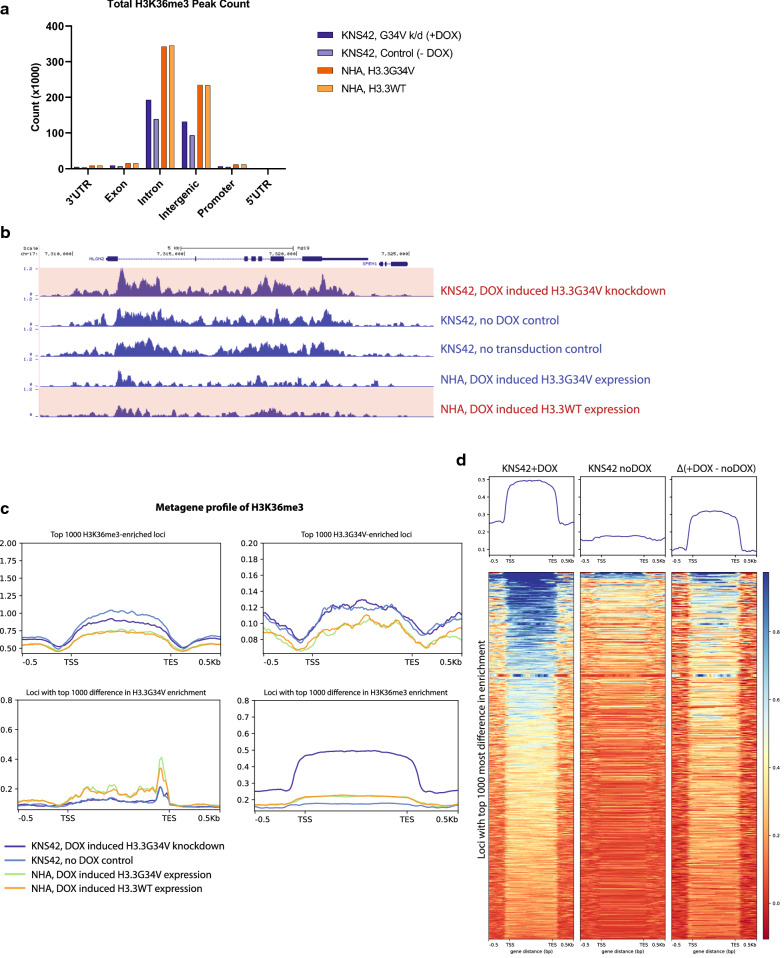
G34V mutation does not affect global H3K36me3. Increased H3K36me3 is observed at loci with G34V knockdown. **a** Relative proportion of H3K36me3 enrichment across gene elements with H3.3G34V expression or knock-down. **b** H3K36me3 enrichment at the *NLGN2* gene is higher in KNS42 after G34V knockdown, compared to KNS42 no DOX control and non-transduced KNS42. However, transduced H3.3G34V into NHAs had no effect on H3K36me3 enrichment patterns. **c** Metagene profile of H3K36me3 enrichment. No significant difference in H3K36me3 enrichment was observed at the 1000 most H3K36me3 enriched loci (*top left*), nor at the 1000 most H3.3G34V enriched loci (top right) with or without DOX-induced H3.3G34V knockdown. No difference H3K36me3 enrichment was also observed at the 1000 loci with the greatest difference in H3.3G34V enrichment between KNS42 no DOX and DOX-induced H3.3G34V knockdown (bottom left). In contrast, greater H3K36me3 enrichment was observed in 1000 loci with the greatest difference in H3K36me3 enrichment with H3.3G34V knockdown, compared to no DOX control (bottom right). **d** Heatmap profiles of K36me3 peaks at loci with the top 1000 most difference in enrichment before and after knockdown. Signals from (left) KNS42 with DOX-induced G34V knockdown, (middle) KNS42 without G34V knockdown (no DOX control), and (left) the difference between them (Δ, left minus middle). Positive values indicate that KNS42 with DOX-induced G34V knockdown has higher K36me3 enrichment compared to no DOX control

Next, we set to determine the effects of H3.3G34V expression on patterns of genomic H3K36me3 enrichment. Consistent with previous studies [17], H3.3G34V knockdown in glioma cells did not result in significant change in the level of global H3K36me3 enrichment across all gene elements evaluated, with a total of 354,857 K36me3 peaks after H3.3G34V knockdown (7187 promoter, 202,284 gene body), compared to 254,315 H3K36me3 peaks cells in KNS42 cells with intact H3.3G34V expression (5193 promoter, 145,856 gene body, Fig. 3a). Similarly, we did not observe a significant difference in the number of H3K36me3 peaks in astrocytes after H3.3G34V knock-in compared to WT cells (Fig. 3a).

In H3.3G34V mutant KNS42 cells, we observed no difference in H3K36me3 enrichment with or without DOX-induced H3.3G34V knockdown, at neither the 1000 most H3K36me3 enriched loci, nor at the 1000 most H3.3G34V enriched loci (Fig. 3c, top left and top right panels). Additionally, we not observe any difference in H3K36me3 enrichment in NHAs, with or without induced H3.3G34V expression. It is possible that H3.3G34V knockdown in KNS42 did not occur at loci with highest H3.3G34V enrichment, nor at loci with highest H3K36me3 enrichment. To test this hypothesis, we examined the loci with highest difference in H3.3G34V and H3K36me3 enrichment, with and without DOX-induced H3.3G34V knockdown. At the 1000 loci with the greatest difference in H3.3G34V enrichment between no DOX and DOX-induced H3.3G34V knockdown conditions, we also saw no difference in H3K36me3 enrichment (Fig. 3c, bottom left panel). Interestingly, at the 1000 loci with the greatest difference in H3K36me3 enrichment with versus without H3.3G34V knockdown, we observed much greater H3K36me3 enrichment with H3.3G34V knockdown compared to no DOX control (Fig. 3c, bottom right panel). For example, we saw reduced enrichment with H3K36me3 at neuroligin-2, *NLGN2* with H3.3G34V knockdown, compared to transduced KNS42 with no DOX control and KNS42 non-transduced cell lines (Fig. 3b). These data are consistent with previous observations that H3.3G34V mutation leads to local reductions in H3K36me3 enrichment. Additionally, our data indicate that the loci with differences in H3K36me3 enrichment after H3.3G34V knockdown are neither those with greatest H3.3G34V enrichment, nor those with the greatest H3K36me3 enrichment.

In contrast, induced expression of H3.3G34V was not sufficient to change genomic H3K36me3 enrichment patterns. Evaluation of the top 1000 loci with the most significantly different H3K36me3 enrichment between no DOX and DOX-induced knockdown of H3.3G34V in KNS42 showed that, compared to no DOX control, KNS42 cells with DOX-induced H3.3G34V knockdown have greater overall H3K36me3 enrichment at 698 loci (Fig. 3d). It has been previously suggested that H3.3G34V expression leads to differential distribution of H3K36me3 on approximately 150 genes [19]. Taken together, our data suggest that DOX-induced H3.3G34V knockdown does not affect the most H3.3G34V or H3K36me3 enriched loci, but does have specific genomic effects on a limited subset of biologically relevant genes.

### H3.3G34V is associated with higher H3K27me3 enrichment

**Fig. 4:**
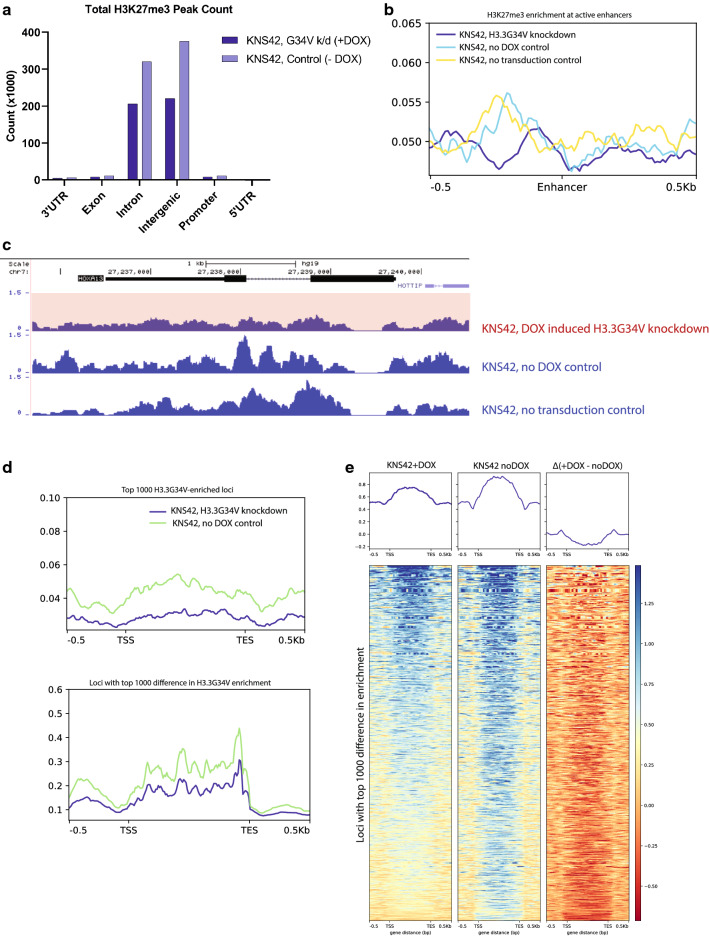
H3.3G34V is associated with higher H3K27me3 enrichment. **a** Relative proportion of H3K27me3 enrichment across gene elements with H3.3G34V expression or knock-down in KNS42 cells. **b** Metagene profile of H3K27me3 enrichment at active enhancers (H3K27ac peaks 2.5 kb away from TSS). Higher H3K27me3 was observed in transduced KNS42 without DOX and non-transduced KNS42 compared to KNS42 with G34V knockdown. **c** At *HOXA13*, H3K27me3 enrichment is lower in KNS42 with G34V knockdown, compared to KNS42 no dox control and non-transduced KNS42. Blue tracks are cell lines that harbor, or overexpressed, H3.3G34V; red tracks are cell lines that do not harbor, or knockdown, H3.3G34V. **d** Metagene profile of H3K27me3 enrichment. H3K27me3 enrichment in the gene body across the top 1000 (top) most G34V-enriched genes in KNS42 TRIP no dox control. (bottom) most diff genes in G34V enrichment before and after KD in KNS42 TIRP ± DOX. **e** Heatmap profiles of K27me3 peaks at the top 1000 most K27me3 enriched loci. Signals from (left) KNS42 with DOX-induced G34V knockdown, (middle) KNS42 without G34V knockdown (no DOX control), and (left) the difference between them (Δ, left minus middle). Negative values in the third column indicate that KNS42 with DOX-induced G34V knockdown has lower K27me3 enrichment compared to no DOX control

Several studies have shown that H3.3G34V directly impacts the modification state of adjacent K27 and K36 residues on the mutant H3.3 protein [2]. In line with this, we observed greater H3K27me3 enrichment in KNS42 cells with H3.3G34V (740,497 peaks total, 11,516 promoter, 332,437 gene body), compared to KNS42 cells after H3.3G34V knockdown (457,844 peaks total, 8270 promoter, 214,405 gene body) (Fig. 4a). The greater enrichment of H3.3K27me3 in non-transduced KNS42 and transduced KNS42 with no DOX control compared to KNS42 cells with DOX-induced H3.3G34V knockdown was observed at active enhancers (Fig. 4b). In turn, at *HOXA13* we saw lower enrichment of H3K27me3 in KNS42 cells with DOX-induced H3.3G34V knockdown, compared to transduced KNS42 with no DOX control and non-transduced KNS42, indicating that the H3.3G34V mutation is associated with higher H3K27me3 enrichment (Fig. 4c). Indeed, at the top 1000 most H3.3G34V-enriched loci in KNS42 without DOX, we observed that KNS42 cells without DOX-induced H3.3G34V knockdown harbored higher HK27me3 enrichment relative to other cell lines studied (Fig. 4d, top panel). This same finding was observed at loci with greatest differential H3.3G34V enrichment between DOX-induced H3.3G34V knockdown cells and no-DOX controls (Fig. 4d, bottom panel). Heatmap profiles of the top 1000 most H3K27me3 enriched loci in KNS42 showed that DOX-induced knockdown of H3.3G34V results in lower relative H3K27me3 enrichment, compared to no DOX controls.

### H3.3G34V enrichment implicates distinct molecular pathways

As H3.3G34V mutation is associated with distinct changes in H3K36me3 and H3K27me3 enrichment patterns, we sought to determine functional pathways of gene expression implicated by differential enrichment patterns of mutant and wild-type H3.3. Functional pathways analysis revealed cellular assembly and organization (*p *= 1.12 × 10^−3^), cellular function and maintenance (*p *= 1.18 × 10^−3^), and cell signaling (*p *= 8.81 × 10^−5^) as the top molecular functions associated with the gene set co-enriched in H3.3G34V and H3K36me3. Cell death and survival (*p *= 1.12 × 10^−2^), cellular development (*p *= 1.25 × 10^−4^), and cellular function and maintenance (*p *= 1.25 × 10^−4^), are the top molecular and cellular functions implicated by the gene set co-enriched in H3.3G34V and K27me3. As expected, the top disease associated with both these gene sets was cancer (*p *= 2.11 × 10^−10^).

GO and KEGG analyses were also performed to identify functional annotations of genes with the most differential H3.3G34V, H3.3WT, and H3K36me3 enrichment between KNS42 with intact H3.3G34V expression and H3.3G34V knockdown. Cellular metabolism and oncogenesis were consistently the top two enriched pathways in cells expressing H3.3G34V (Additional file 2: Figure S2), with metabolic pathways implicated as the top cellular function of this gene set (−log(*p* value) = 12.92–21.15, Additional file 3 Figure S3). Cellular neuron projection morphogenesis and neuron differentiation were also highly enriched in this gene set, consistent with previous studies showing genes enriched in H3K36me3 contribute to in neuronal differentiation and cell proliferation [19].

## Discussion

Somatic missense mutations that alter histone H3.3 structure and function are uniquely common in pediatric high-grade glioma. Tumors harboring the H3.3G34V/R mutation are clinically and biologically distinct from wild type tumors, with poorer progression free and overall survival, and unique genomic, proteomic, methylomics and epigenetic profiles relative to wild-type tumors [5, 9]. However, the mechanisms by which these mutations lead to tumorigenesis are still not completely understood. While prior studies have attempted to compare the effects of H3.3G34V in vitro, these studies did not employ isogenic cell lines. Here, we genetically modified pediatric glioma cells and normal astrocytes using a DOX-inducible construct in order to more accurately determine the effects of H3.3G34V expression on the glioma epigenetic landscape that may contribute to tumorigenesis.

Prior studies have shown that unlike the K27 and K36 amino acid residues on the histone H3 N-terminal tail, the H3G34 residue is not post-translationally modified. However, H3G34 does lie in close proximity to H3K36, which undergoes methylation during transcriptional elongation. As a result, H3.3G34V/R mutations may alter accessibility of H3K36 to lysine methyltransferases, thereby affecting H3K36 methylation and hence gene expression. For example, a recent study reported H3.3G34V/R mutations are associated with altered H3K36 and H3K27 methylation on the same mutated histone tails (*in cis*), rather than the global methylation changes observed in association with H3K27M mutations [17]. H3.3G34V/R mutations are also thought to impair the catalytic activity of STED2 and other histone methyltransferases, by substituting G34 with a large side chain residue arginine (G34R) or valine (G34V), preventing H3K36 from fitting the methyltransferase catalytic domain and thereby reducing H3K36 methylation [4, 7]. These large H3.3G34V/R mutant side chains also prevent H3K36 from interacting with DNA mismatch repair protein MutSα, leading to genome instability and tumorigenesis [4]. Here, we sought to better identify the effect of H3.3G34V expression on genomic H3K36 and H3K27 modification states and enrichment patterns.

First, we observed global H3.3G34V co-enrichment with wild type H3.3, suggesting that H3.3G34V may be incorporated with H3.3WT in heterotypic nucleosomes. In addition, the observed residual wildtype H3.3 after *H3F3A* knockdown provides insight into the relative proportion of *H3F3A* and *H3F3B* expression in H3.3G34V mutant tumors. As both *H3F3A* and *H3F3B* encode H3.3 with identical amino acid sequences, it is not possible to distinguish which isoform was expressed based on observed H3.3 protein levels. In contrast, the H3.3G34V mutation only occurs on *H3F3A,* so the relative proportion of wild type and mutant protein after *H3F3A* knockdown can indicate how much H3.3 is derived from expression of each isoform. Indeed, we observed decreased H3.3G34V but little change in wildtype H3.3 levels with DOX-induced *H3F3A* knockdown, indicating the majority of observed H3.3 protein expression is due to *H3F3B* expression in this cell line.

In addition, our western blot analysis demonstrated that H3.3G34V knockdown does not alter global H3K36me3 levels. This result is not surprising: while we observed mutant *H3F3A* knockdown efficiency of 80% via FACS analysis, the H3.3G34V mutation only arises on a single *H3F3A* allele, with a maximum potential allelic frequency of 50%, limiting the starting amount of H3.3G34V mutant protein, prior to knockdown. In addition, the direct effects of H3.3G34V on H3K36 methylation is limited to only the *cis* arm. Therefore, to investigate the effect on H3.3G34V knockdown on H3K36me3 *in cis*, we evaluated H3K36me3 enrichment on loci with residual H3.3G34V after knockdown, and found that these loci were not those with the greatest change H3K36me3 enrichment across the genome. We then focused on the 1000 loci with the greatest relative change in H3K36me3 enrichment after H3.3G34V knockdown, and found that less H3K36me3 enrichment at these loci with intact H3.3G34V expression, consistent with previous findings that G34V is associated with reduction of H3K36me3 [4, 7]. Importantly, we found that knocking down H3.3G34V in the same glioma cell line is sufficient to restore H3K36me3 at these loci. While this observation has been previously reported, our study is the first demonstrate this finding using a controlled transgenic mechanism in an isogenic glioma cell line. This observation is consistent with the theory that the G34 residue is involved in histone H3 recognition by SETD2, which has a binding pocket for G34 so small only glycine may fit inside. As a result, any amino acid substitution on G34 would abolish H3 recognition of by SETD2, thus reducing H3K36me3 on that particular histone protein [19]. Of note, while we did observe differential H3K36me3 enrichment between H3.3G34V glioma cells and the same cell line after G34V knockdown, we were not able to recapitulate this finding by inducing H3.3G34V expression in NHAs. This result suggests that ectopic H3.3G34V expression alone is not sufficient to render the observed epigenetic effects of the mutant protein.

Lastly, we observed greater genomic H3K27me3 enrichment in the presence of H3.3G34V mutant protein. This, and our observation that the H3K36me3 is reduced in the presence of H3.3G34V, is again consistent with previous findings that H3K27me3 rarely co-exists with H3K36me3 on the same histone H3 polypeptide [8]. Previous findings have identified H3K36 methylation as a chromatin component that restricts the PRC2-mediated spread of H3K27 methylation [22]. Our study is the first to support the role of H3K36 methylation in antagonizing PRC2-mediated H3K27 methylation using an isogenic DOX-inducible system in a H3.3G34V mutant glioma cell line, allowing us to attribute the observed changes in H3K36me3 and K37me3 to the presence or absence of the H3.3G34V mutant protein. Our observation is in line with recent studies that also report increased H3K27me3 and decreased H3K36me3 when H3.3G34W is expressed in mesenchymal stem cells [7], as well as in H3.3G34W mutant giant cell tumor of bone (GCT) cell lines [8]. Together, these findings are in line with structural studies that proposed substitutions at H3.3G34 residues (H3.3G34V/W) obstruct STED2 activity via similar mechanisms, by generating steric hindrance to SETD2 activity [7, 21]. In turn, obstruction of SETD2 activity gives rise to changes in H3K27me3 and H3K36me3 levels. Functional pathways analyses of differentially enriched gene sets further validated that our approach recapitulates previous findings, but in a more precise and controlled manner, and underscore the role of H3.3G34V in tumorigenic patterns of gene expression that are biologically relevant.

While our use of isogenic cell lines for these studies is novel and increases our understanding of the epigenetic effects of the G34V mutation, this study is not without limitations. For example, lentiviral-mediated shRNA knockdown and cDNA expression may not be as robust as gene editing techniques. Specifically, our approach does not generate a complete knockdown of the G34V mutation, nor does it introduce similar levels of G34V mutant protein in NHA cells compared to KNS42 cell lines. This could explain why we observed no significant differences in cell viability and proliferation between KNS42 with and without G34V knockdown and between NHA with H3.3 G34V and H3.3 control overexpression. In fact, in CRISPR/Cas9-mediated knockdown of H3.3G34W, significant decrease in proliferation and colony formation was observed in G34W knockout clones [8]. Therefore, employing a more precise method such as CRISPR/Cas9 would be a reasonable next step to further compare the effects of mutant H3.3G34V expression in these isogeneic cell models. Additionally, we also acknowledge that comparing glioma cell line KNS42 to normal human astrocytes (NHAs) is not without caveats. Indeed, there are inherent differences between tumor and normal cell lines, while differences in culture conditions can influence their morphology and observed epigenetic changes. Therefore, it is expected that the observed changes in H3K36me3 and H3K27me3 enrichment in KNS42 with and without DOX-induced G34V knockdown will not be fully recapitulated in NHA with and without G34V overexpression. However, we chose to overexpress H3.3G34V in NHA’s because of the stable astrocytic nature of KNS42 over time. Indeed, the astrocytic nature of KNS42 was confirmed in culture by immunohistochemistry with glial markers GFAP, vimentin, and S100 in 1987 [18] and again in 2009 [1]. This consistency led us to choose wild-type NHA’s to perform these comparative experiments. Lastly, while we identified changes in genomic enrichment patterns of H3.3, H3K27me3 and H3K36me3, our data do not address how the G34V mutation affects higher order chromatin structure, as is observed in K27M mutations [13]. As are all in vitro studies, the methodology of changes to cells may affect the expression level of oncoproteins different from the pathophysiological conditions. Based on our findings, further study into these effects is warranted.

## Conclusions

In summary, our isogenic, DOX-inducible shRNA knockdown of *H3F3A* is a powerful tool that allowed us to investigate the effect of H3.3G34V mutant protein on neighboring H3K36me3. Consistent with previous studies, we observed a decrease in H3K36me3 enrichment in specific genomic loci with H3.3G34V knockdown, and increase in H3K27me3 enrichment. Our data demonstrate that H3.3G34V knockdown is sufficient to reverse the effects of the G34V mutation on attenuating the function of SETD2 to methylate H3K36 at selected loci, but does not affect global H3K27 or H3K36 methylation levels. Further investigation into the epigenetic effects of the H3.3G34V mutation are therefore warranted, as better understanding of how this mutation drives pediatric gliomagenesis may be valuable for identifying more rational, effective therapeutic targets for improved treatment of pediatric high-grade glioma.

## Supplementary Information

**Additional file 3: Figure S1.** Characterization of pediatric glioma cells and astrocytes with genetic modification of histone H3.3G34V expression. A) Relative proportion of H3.3G34V enrichment across gene elements in non-transduced KNS42 or NHA with DOX-induced H3.3G34V overexpression or H3.3 expression control. B) H3.3G34V protein level in non-transduced KNS42 or NHA with DOX-induced H3.3G34V overexpression or H3.3 expression control. H3.3G34V protein expression was observed to be 29% of that expressed in non-transduced KNS42. C) Cell viability in modified cell lines. No significant difference was observed in cell viability between KNS42 with G34V knockdown, and KNS42 with no doxycycline control, as well as in NHA overexpressed with H3.3G34V and overexpression control, NHA overexpressed with H3.3. *X*-*axis*: absolute luminescence. *Y*-*axis:* days following plating. Error bars represent standard error. D) Rate of cell proliferation relative to control. No significant difference was observed in cell proliferation in KNS42 with G34V knockdown compared to no doxycycline control. NHA overexpressed with H3.3G34V showed lower, but not significant, proliferation compared to NHA with overexpression control. *X*-*axis*: proliferation rate normalized to control. *Y*-*axis*: days following plating. D) Light microscopy images of cells at 10x magnification on days 3 and 7 after plating.

**Additional file 3: Figure S2.** GO and KEGG analysis of genes with the most differentially enriched A) H3.3G34V, B) H3.3WT, and C) H3K36me3 in KNS42 vs KNS42 with H3.3G34V knockdown. Y-axis represents the enriched GO biological process and KEGG pathways, X-axis represents the number of unique genes enriched. Statistical significance determined via FDR < 0.05, adj *p *< 0.05.

**Additional file 3: Figure S3.** KEGG analysis of gene co-enriched in G34V and H3.3 (*top*) and co-enriched in G34V and K36me3 (*bottom*). Number is −log(*p*-value).

## Data Availability

ChIP-Seq data generated during the current study are available on Mendeley Data under 10.17632/hh6x2fjkvd.1 (https://data.mendeley.com/datasets/hh6x2fjkvd/draft?a=50637383-2896-41a6-9304-469e438b0aa5). All remaining data generated and/or analyzed are included in this published article and associated supplemental data files.

## Abbreviations

DOX: doxycycline
WT: wild type
pHGG: pediatric high-grade glioma
ChIP-Seq: chromatin immunoprecipitation with massively parallel DNA sequencing
GO: gene ontology
KEGG: Kyoto Encyclopedia of Genes and Genomes
