# Supplementary figures and images for: Effects of H3.3G34V mutation on genomic H3K36 and H3K27 methylation patterns in isogenic pediatric glioma cells
Source: Acta Neuropathol Commun. 2020 Dec 7;8:219. doi: 10.1186/s40478-020-01092-4 (PMC7722426; doi:10.1186/s40478-020-01092-4)

A

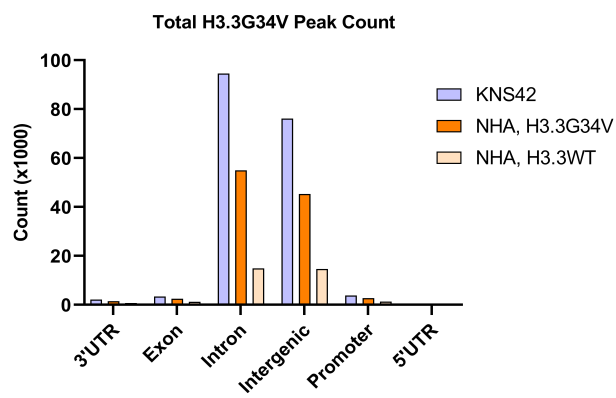

B

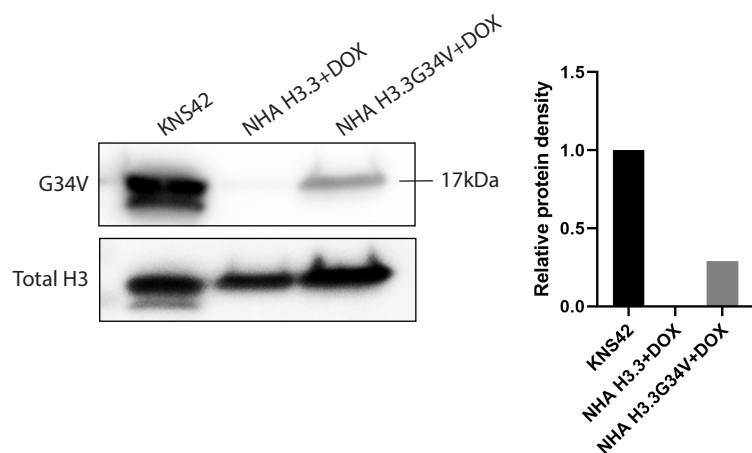

C

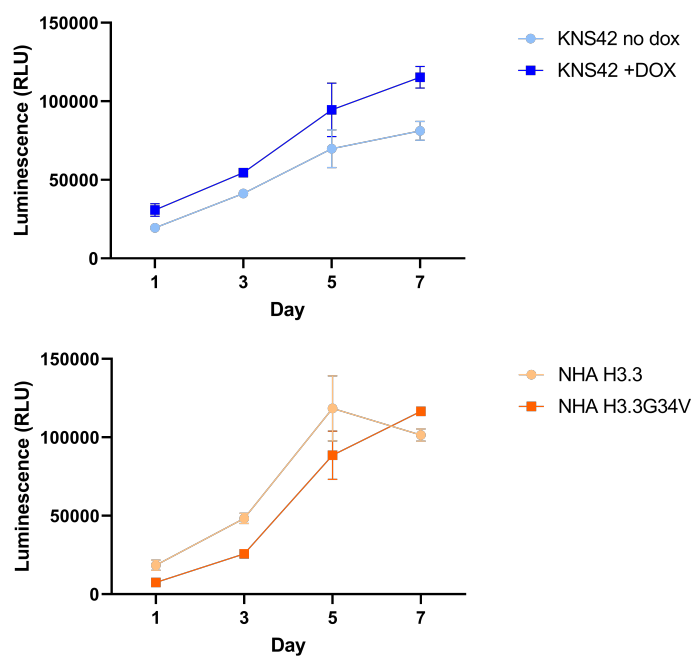

D

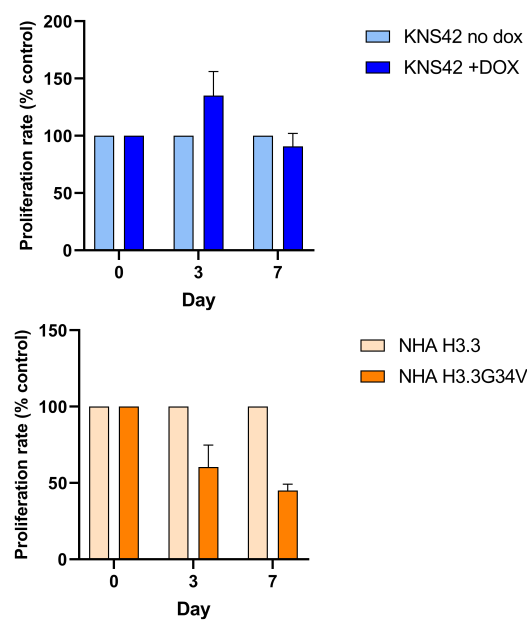

E

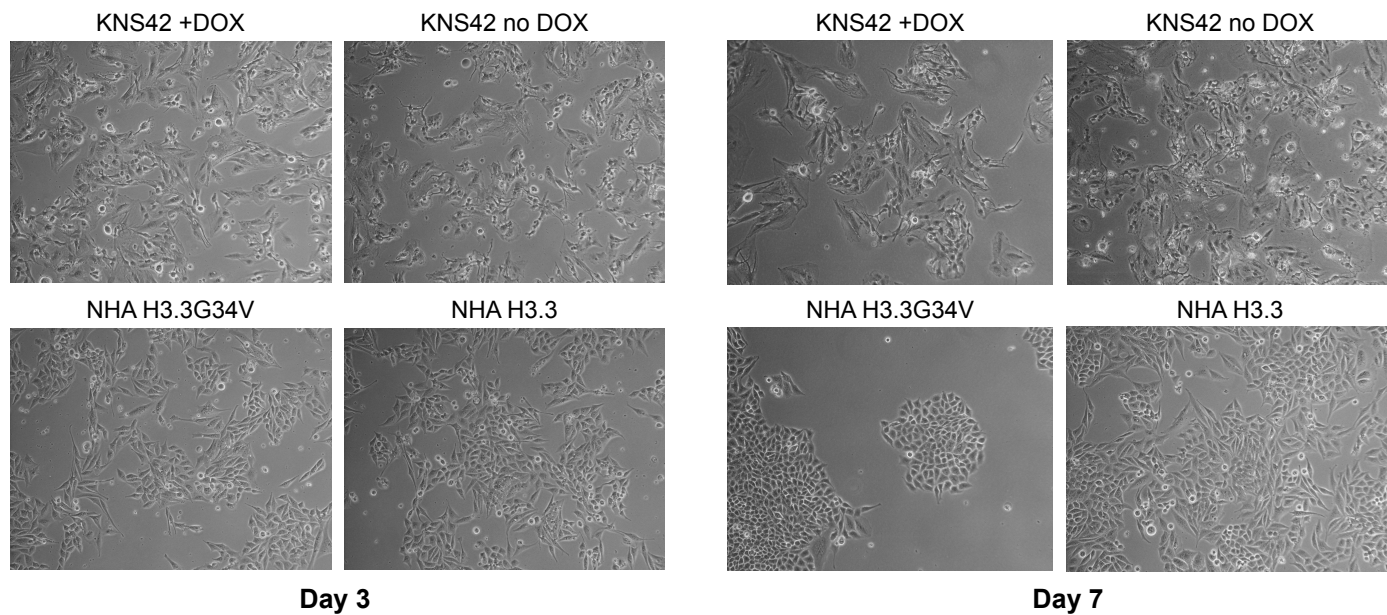

Supplement: Supplementary file 1 — Additional file 3: Figure S1. Characterization of pediatric glioma cells and astrocytes with genetic modification of histone H3.3G34V expression. A) Relative proportion of H3.3G34V enrichment across gene elements in non-transduced KNS42 or NHA with DOX-induced H3.3G34V overexpression or H3.3 expression control. B) H3.3G34V protein level in non-transduced KNS42 or NHA with DOX-induced H3.3G34V overexpression or H3.3 expression control. H3.3G34V protein expression was observed to be 29% of that expressed in non-transduced KNS42. C) Cell viability in modified cell lines. No significant difference was observed in cell viability between KNS42 with G34V knockdown, and KNS42 with no doxycycline control, as well as in NHA overexpressed with H3.3G34V and overexpression control, NHA overexpressed with H3.3. X-axis: absolute luminescence. Y-axis: days following plating. Error bars represent standard error. D) Rate of cell proliferation relative to control. No significant difference was observed in cell proliferation in KNS42 with G34V knockdown compared to no doxycycline control. NHA overexpressed with H3.3G34V showed lower, but not significant, proliferation compared to NHA with overexpression control. X-axis: proliferation rate normalized to control. Y-axis: days following plating. D) Light microscopy images of cells at 10x magnification on days 3 and 7 after plating. [file 40478_2020_1092_MOESM1_ESM.pdf]

### Unique genes co-enriched in G34V and H3.3

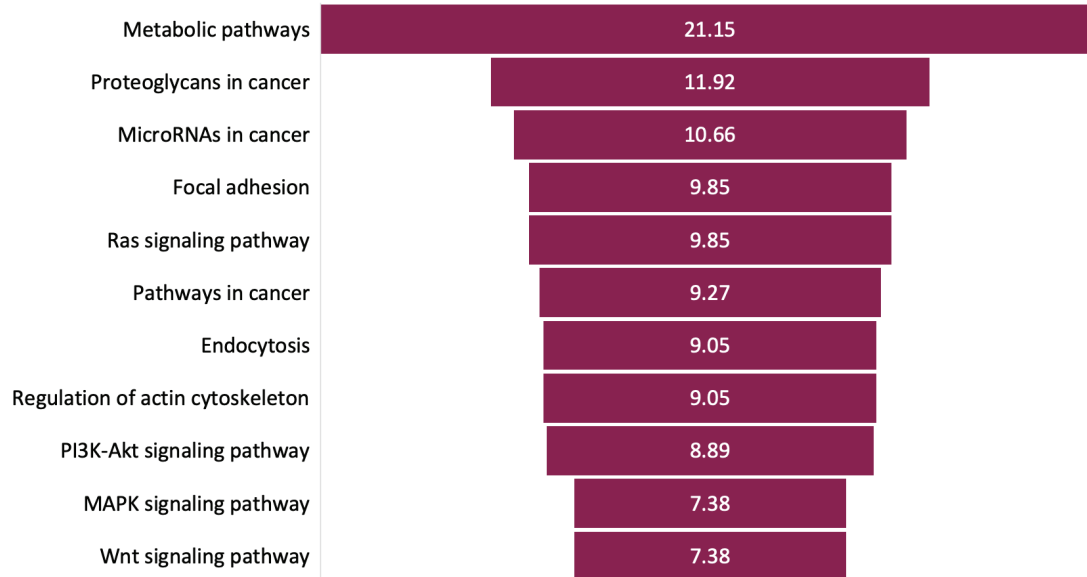

### Unique genes co-enriched in G34V and K36me3

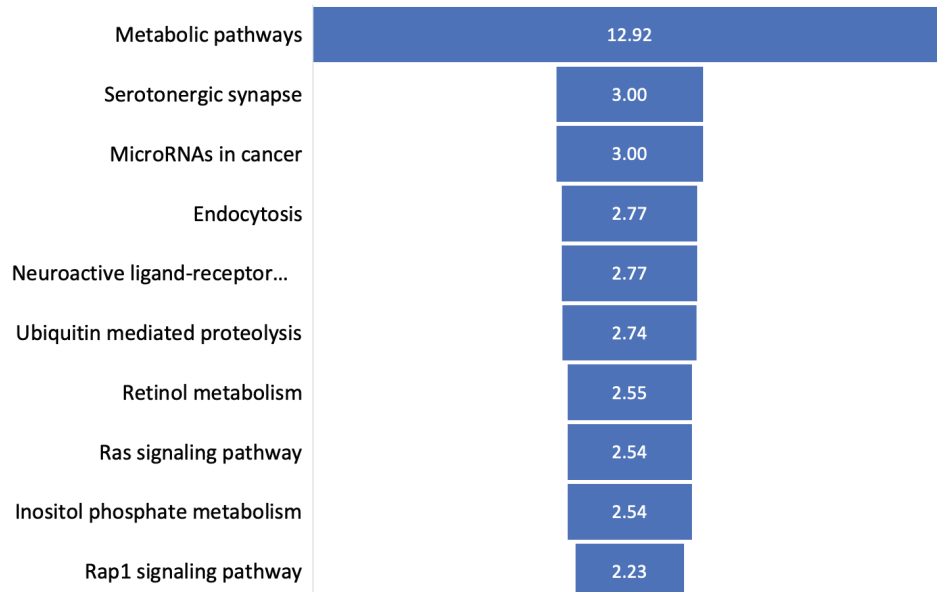

Supplement: Supplementary file 3 — Additional file 3: Figure S3. KEGG analysis of gene co-enriched in G34V and H3.3 (top) and co-enriched in G34V and K36me3 (bottom). Number is −log(p-value). [file 40478_2020_1092_MOESM3_ESM.pdf]
