# Supplementary material for: Effects of H3.3G34V mutation on genomic H3K36 and H3K27 methylation patterns in isogenic pediatric glioma cells
Source: Acta Neuropathol Commun. 2020 Dec 7;8:219. doi: 10.1186/s40478-020-01092-4 (PMC7722426; doi:10.1186/s40478-020-01092-4)
Supplement: Supplementary file 2 — Additional file 3: Figure S2. GO and KEGG analysis of genes with the most differentially enriched A) H3.3G34V, B) H3.3WT, and C) H3K36me3 in KNS42 vs KNS42 with H3.3G34V knockdown. Y-axis represents the enriched GO biological process and KEGG pathways, X-axis represents the number of unique genes enriched. Statistical significance determined via FDR < 0.05, adj p < 0.05. [file 40478_2020_1092_MOESM2_ESM.pdf]

GO Biological Process

KEGG Pathway

Most differentially enriched H3.3G34V loci

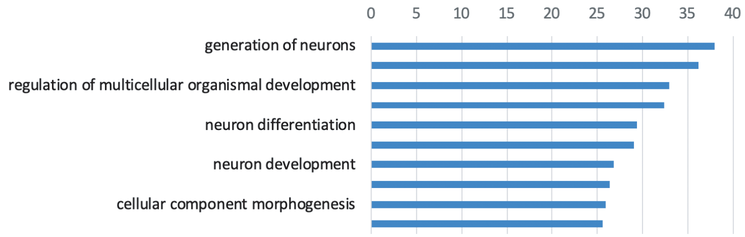

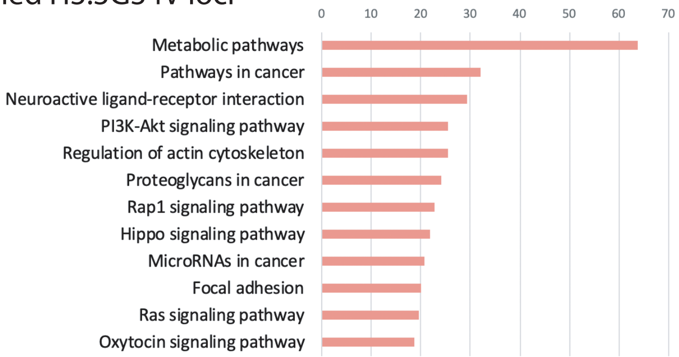

Most differentially enriched H3.3WT loci

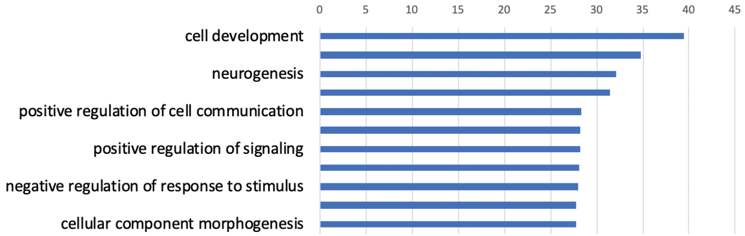

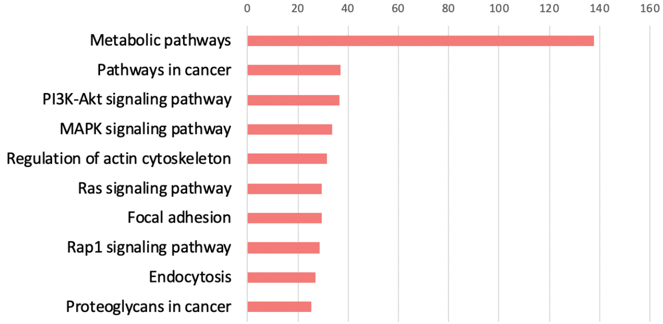

Most differentially enriched H3K36me3 loci

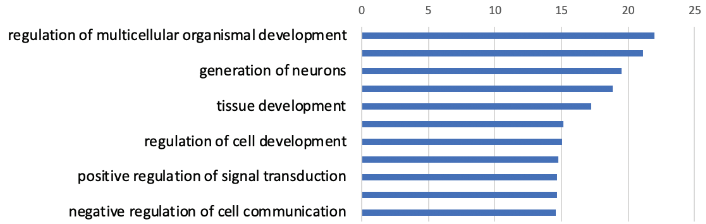

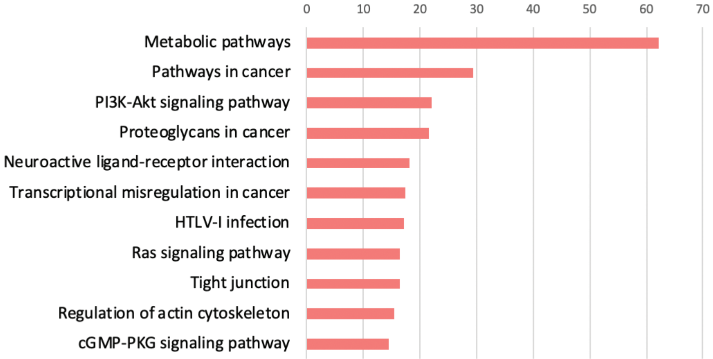

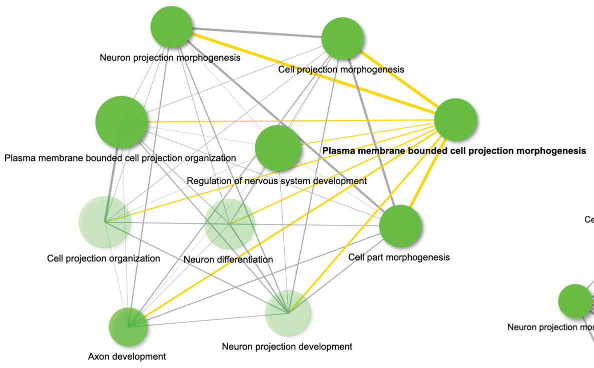

H3.3WT

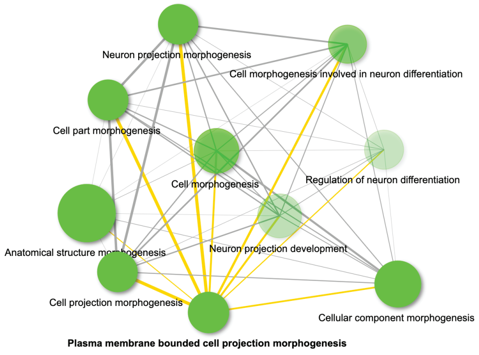

H3.3G34V

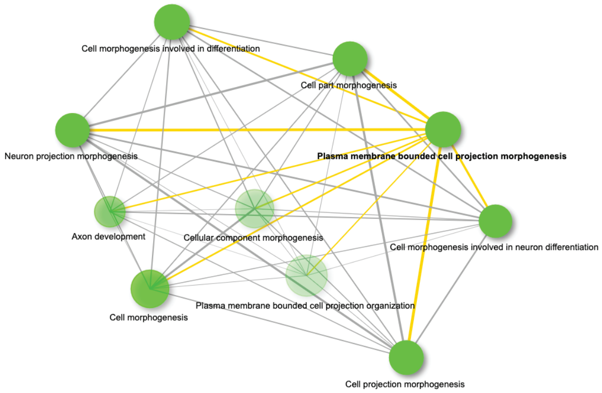

H3K36me3
